# Effects of branched chain amino acids, l-citrulline, and alpha-glycerylphosphorylcholine supplementation on exercise performance in trained cyclists: a randomized crossover trial

**DOI:** 10.1080/15502783.2023.2214112

**Published:** 2023-05-25

**Authors:** Renee Nicole Harrington

**Affiliations:** Food, Bioprocessing and Nutrition Sciences, North Carolina State University, Raleigh, NC, USA

**Keywords:** Performance, endurance exercise, power output, perceived exertion, fatigue, time trial

## Abstract

**Background:**

Nutrition plays a key role in training and athletic performance and dietary supplements can make a small, but potentially valuable, contribution to achieving peak athletic performance. This study is the first to investigate the effects of supplementation from the combination of BCAAs, L-citrulline, and A-GPC on exercise performance.

**Methods:**

In this randomized, double-blind, crossover study 30 male trained cyclists (age: 43.7 ± 8.5 years) completed a 20 km cycling time trial (TT) test and a high intensity endurance cycling (HIEC) test following a 7-day supplementation period with either a supplement containing 8 g BCAAs, 6 g L-citrulline, and 300 mg A-GPC or a placebo (15 g maltodextrin). For each trial, mean values for time to completion, peak and average power output, OMNI rating of perceived exertion, and visual analogue scale (VAS) responses on perceived exertion were computed for the 20 km TT test. Mean values for time to fatigue and VAS responses on perceived exertion were computed for the HIEC test. Procedures for dietary intake and exercise patterns were implemented to achieve consistency throughout the study period.

**Results:**

There was a significant increase (*p* = .003) in peak power in the 20 km TT (354.27 ± 87.88 and 321.67 ± 63.65, for supplement and placebo trials, respectively) and a significant increase (*p* = .001) in time to fatigue in the HIEC test (0:19:49 ± 0:11:13 min and 0:14:33 ± 0:09:59 min, for supplement and placebo trials, respectively) with the test supplement compared to the placebo. With the test supplement, there was an average increase in TT peak power of 11% and an average increase in time to fatigue of 36.2% in the HIEC test compared to the placebo. There was no significant improvement in time to completion, average power, OMNI rating of perceived exertion, or VAS responses on perceived exertion in the TT test and no significant improvement in VAS measures of perceived exertion in the HIEC test.

**Conclusions:**

The combination of BCAAs, L-citrulline, and A-GPC used in this study improves cycling performance and may be useful for individuals seeking to improve athletic performance, particularly in disciplines requiring lower body muscular strength and endurance.

## 1. Background

Exercise-induced muscular fatigue is an unavoidable effect that can limit performance and accomplishment of training goals and thus has been a focus of research in sports and exercise. Fatigue can be categorized as either peripheral or central. Peripheral fatigue results from a number of aspects including accumulation of metabolites, protein breakdown, and carbohydrate consumption; whereas central fatigue is primarily the result of increasing levels of the neurotransmitter serotonin (5-hydroxytryptamine; 5-HT) that results in the feeling of total body fatigue (i.e. lethargy, tiredness, and loss of motivation) [1,2]. It is suggested that nutritional interventions that improve both central and peripheral adaptations are important for achieving the optimal performance [3].

Amino acids have been the focus of research for many years for their ability to enhance athletic performance by modifying fuel utilization during exercise, preventing fatigue, and improving recovery. BCAAs are unique among the essential amino acids for their role in numerous metabolic pathways that affect exercise [1] and are theorized to enhance performance in a variety of ways, including having beneficial impacts on measures of muscle protein synthesis and breakdown [4], central fatigue [5–7], muscle soreness, rate of perceived exertion, and recovery [1]. However, one disadvantage to BCAA supplementation is the excess levels of ammonia that results from increased BCAA metabolism during exercise. Research has shown that ammonia contributes to both central and peripheral fatigue [8,9], and thus would nullify the potential benefit of BCAA supplementation on physical and cognitive performance [10].

Arginine and citrulline are two amino acids that research has shown to reduce exercise-related accumulations of ammonia by promoting the urea cycle and nitric oxide (NO) biosynthesis. Research suggests that combining arginine and citrulline with BCAA supplementation may alleviate the excess ammonia produced during exercise and BCAA catabolism [10]. In addition, NO is a blood vessel dilator and contributes to increased blood flow, oxygen, and nutrient delivery to active muscles [11]. More recent literature has indicated that citrulline is a more potent NO precursor than arginine due to its high bioavailability [12] and ability to enhance circulating arginine levels more effectively than oral arginine supplementation [13,14].

Choline is an essential nutrient that has been a focus of recent research for improved performance as well. As a water-soluble quaternary amine of the vitamin B group, choline is important for a number of physiological pathways [15]. Research suggests that strenuous exercise may stress several pathways and can create a significant, short-term decrease in free (non-membrane-bound) choline in the blood. Muscular performance would then be inhibited by decreased availability of choline for acetylcholine synthesis, impacting excitation – contraction coupling at the neuromuscular junction [16]. It is also suggested that choline supplementation can improve performance by having a beneficial impact on mental focus and reaction time [17]. Alpha-glycerylphosphoryl choline (A-GPC), a molecule in the choline synthesis pathway, has been receiving recent praise as a performance-enhancing supplement for physical and mental function. A-GPC is a semi-synthetic derivative of lecithin, which releases free choline that can be synthesized into acetylcholine and phosphatidylcholine. Research suggests that A-GPC may be effective in increasing peak power and delaying neuromuscular fatigue in athletes [3,18].

The current study examined the effectiveness of chronic (7-day) supplementation of BCAAs, L-citrulline, and A-GPC on physical performance in trained cyclists. It was theorized that the supplement would result in improved performance by increasing peak and average power, reducing time to completion, and reducing measures of perceived exertion in a 20 km TT test and increasing time to fatigue and reducing measures of perceived exertion in a HIEC test as compared to a placebo.

## 2. Materials and methods

### 2.1. Participants

Thirty male trained cyclists (age: 43.7 ± 8.5 years; weight: 79.2 ± 9.6 kg; body fat: 19.2 ± 5.3%) were recruited. Inclusion criteria included: male, 30–60 years old, minimum of 5 years of consistent cycling training (i.e. cycling on average 5+ hours weekly). Exclusion criteria included: taking medication or steroids to enhance physical performance; a weight loss of >5 kg in the previous 3 months; history of drug abuse, smoking 10+ cigarettes a day or consuming more than 14 g of alcohol daily (1.4 standard drinks); orthopedic injury, or surgery within previous 6 months; any other condition judged problematic for participation in the study.

Participants were instructed to maintain regular physical activity (i.e. engage in aerobic exercise at or above 75% maximum intensity for 45–60 min/day; 4+ days/week) throughout the study; however, they were instructed to abstain from intense exercise for 72 h prior to each testing session, refrain from exercise 24 h before each testing session, and to arrive rested and hydrated. Participants were instructed to maintain normal dietary patterns but abstain from energy drinks and ergogenic substances throughout the study. Participants were given a diet and activity log to record food intake and exercise during the 72 h prior to the first testing session and were instructed to repeat the diet and activity pattern for the second trial.

### 2.2. Study procedures

#### 2.2.1. Study design and supplementation regimen

The Institutional Review Board at the North Carolina State University reviewed the present investigation for ethics. The study was a randomized, double-blind, crossover with a 7-day washout period. Participants completed a preliminary familiarization session (day 0), followed by ingestion the next day (day 1) of either supplement or placebo as provided. On days 1–6 of supplementation, participants ingested the supplement in the morning with breakfast, and on day 7 (the experiment day) they ingested the supplement 1 h prior to completing the exercise protocol. This timing is consistent with literature and data indicating a peak concentration approximately 1 h after ingestion [12]. The participants had a washout period of 7 days before receiving the next package of supplement and beginning the second treatment cycle. All testing occurred at the same time of day (±1 hr) to avoid the effects of circadian rhythm on physical and mental performance [19,20]. Testing was conducted in a climate-controlled room, and equipment was calibrated before each session.

The supplement and placebo (see Table 1 for the formulations) were provided in individual packets of ready-to-mix powder to be dissolved in 16–20 ounces of water. The active treatment consisted of 8 g of BCAAs (2:1:1 ratio of leucine, isoleucine, valine), 6 g of L-citrulline, and 300 mg of A-GPC. The level of these ingredients is comparable to the dosage used in previous studies demonstrating effectiveness [7,17,21–23]. Maltodextrin was used as a placebo as it has a very similar appearance and no influence on the target outcomes evaluated [24,25]. Both the test supplement and placebo contained B-vitamins and electrolytes for functionality and citric acid for stabilization of ingredients. The test supplement also contained a bitterness masker. The appearance, weight, smell, and taste of the supplement and placebo powders were confirmed to be indistinguishable by the manufacturer of the supplements (Allen Flavors Inc., South Plainfield, NJ; Ajinomoto, Raleigh, NC).

**Table 1.** Supplement and placebo formulation.

Supplement		Placebo	
Ingredient Description	Amount (g)	Ingredient Description	Amount (g)
BCAAs (2:1:1)^1^	8.4^2^	Maltodextrin	14.952
L-Citrulline	6.2^2^		
A-GPC	0.312^2^		
Lemon Lime flavor	1.2	Lemon Lime flavor	1.2
Steviol Glycosides	0.5	Steviol Glycosides	0.5
Citric Acid	2.0	Citric Acid	2.0
Potassium Citrate	0.207	Potassium Citrate	0.207
Sea Salt	0.582	Sea Salt	0.582
Thiamin (Vitamin B1) (100%DV)	0.0018	Thiamin (Vitamin B1) (100%DV)	0.0018
Riboflavin (Vitamin B2) (25%DV)	0.0005	Riboflavin (Vitamin B2) (25%DV)	0.0005
Niacinamide (Vitamin B3) (100%DV)	0.0245	Niacinamide (Vitamin B3) (100%DV)	0.0245
D-Calcium Pantothenate (Vitamin B5) (100%DV)	0.011	D-Calcium Pantothenate (Vitamin B5) (100%DV)	0.011
Pyridoxine Hydrochloride (Vitamin B6) (200% DV)	0.0041	Pyridoxine Hydrochloride (Vitamin B6) (200% DV)	0.0041
Biotin (Vitamin B7) (100%DV)	0.0054	Biotin (Vitamin B7) (100%DV)	0.0054
Folic Acid (Vitamin B9) (50%DV)	0.0017	Folic Acid (Vitamin B9) (50%DV)	0.0017
Cyanocobalamin (Vitamin B12) (500%DV)	0.0024	Cyanocobalamin (Vitamin B12) (500%DV)	0.0024
Bitterness masker	1.2		
Total	20.69^3^	Total	19.49^3^

1.2:1:1 ratio of leucine, isoleucine, valine

2.Overage included to ensure target amounts were met.

3.Negligible difference in total weights due to absence of bitterness masker in the placebo.

#### 2.2.2. Preliminary familiarization session

Participants completed a 10-min familiarization ride to allow habituation to the laboratory equipment and procedures employed in the study. Participants then completed a Maximal Workload Test (Wmax) followed by the remaining tests at submaximal levels for familiarization. Participants adjusted the cycle ergometer seat and handlebar heights and distances that they felt best matched their own personal bike setup. Bike set-up measurements were recorded and kept consistent for all performance testing sessions. Participants were allowed to use their own cycling shoes for all testing if desired.

#### 2.2.3. Astrand-rhyming cycle ergometer test to determine maximal workload

The subject pedaled with resistance and cadence of choice for 6 min at a workload that elicited a steady-state heart rate between 125 and 170 bpm. If a steady-state heart rate in that range was not achieved, the subject adjusted the workload appropriately and continued for a second 6-min period. Otherwise, testing was complete. Heart rate was recorded every minute during the test. Predicted VO_2_ (L/min) was calculated with the modified Astrand-Rhyming nomogram [26,27].

Participants were then directed to maintain 80–90 revolutions per minute (RPM) while cycling at a wattage (W) equivalent to 3.25 * body weight (kg). Wattage was increased 25 W every 2.5 min until reaching volitional exhaustion, which was defined as when the subjects indicated they could not continue exercising at the intensity or they were unable to maintain the wattage. Subjects were instructed to complete the entire test seated to prevent use of body mass momentum for added power output.

To equalize differences in exercise capacity and weight (i.e. power to body weight ratio), starting wattage was increased to either 3.5, 3.75, or 4 * body weight (kg) when the calculated starting wattage was below the average wattage maintained in the Astrand-Rhyming test. When increased, the starting wattage was chosen to be between 50 and 100 W of the average wattage maintained in the Astrand-Rhyming test, with consideration of the participant’s heart rate and reported perceived exertion, and such that each participant would complete the Wmax test within 10 min following protocol by Arts et al. [28].

Maximal workload (Wmax) was calculated as follows: Wmax= Wf + [(t/D × P)], where Wf is the power output during the last completed stage, t is the duration of the last uncompleted stage, D is the duration of each stage in seconds (= 150 s), and P is the incremental increase in power output with every stage (= 25 W) [1,29].

#### 2.2.4. Performance testing sessions

At each testing session, anthropometric measurements were taken (weight, BMI, and body fat %) using a medical grade scale (InBody H20N; Cerritos, California) and participants warmed-up for 10 min on an electromagnetically braked cycle ergometer at a speed and resistance of their choice. Participants then completed a self-paced 20 km cycling TT, followed by a 10-min recovery to return heart rate to baseline, and finally a HIEC test. Participants had ad libitum access to plain water during the first trial. Fluid intake was recorded and matched during the subsequent trial.

##### 2.2.4.1. 20 km time trial test

Participants completed the individualized total work in the shortest time at a self-selected resistance and pedaling cadence. Subjects were instructed to complete the entire test seated to prevent use of body mass momentum for added power output and were directed to maintain their normal cadence. Participants received no verbal encouragement and no physiological feedback other than the distance covered at 5 km, 10 km, 15 km, and 20 km. Peak and average power output, and time to completion were recorded at each distance mileage mark and participants were asked to indicate perceived exertion using the OMNI-cycle scale [30] that was in view during the test. Performance outcomes that subjects were able to see on the ergometer display screen included distance covered, gear selection, RPM, and instantaneous power. Participants were blinded to all other measures. Upon completion of the test, participants were asked to provide responses to a visual analogue scale (VAS) questionnaire regarding their perception of physical exertion (muscular fatigue, muscular soreness) and other subjective conditions (overall energy, ease of pedaling, mental focus/concentration). This 0 (best outcome) to 100 (worst outcome) scale has been used previously to assess musculoskeletal fatigue [31].

##### 2.2.4.2. HIEC test

Using modified protocol outlined in the literature [1,32], participants completed a HIEC test using Wmax determined during the preliminary familiarization session. Participants completed an initial 2-min warm-up period to build up to the starting workload, followed by four 2.5-min continuous progressive increments of cycling at a workload corresponding to 70%, 75%, 80%, and 85% of Wmax. Participants then completed ten 90-s sprints at a workload corresponding to 90% Wmax, separated by 180-s recovery at a workload corresponding to 55% Wmax. If the participant completed all 10 sprints, following a three-minute interval at 55% Wmax, a capacity trial to fatigue was undertaken at 90% Wmax. Exhaustion during the test was defined as an inability to maintain power output within 5 W of expected and an inability to restore within 15 s despite verbal encouragement. No feedback on elapsed time was provided so as not to introduce potential bias with participants targeting previous times. Participants were asked to either raise their hand or stop pedaling to indicate exhaustion so distance and time to exhaustion could be recorded instantly. Participants were instructed to complete the entire test seated to prevent use of body mass momentum for added power output. Upon completion of the test, participants once again provided responses to the VAS questionnaire. Gear selection and RPM for the specific power outputs were recorded during the initial trial and replicated the second trial. Performance outcomes that subjects were able to see on the ergometer display screen included gear selection, RPM, and instantaneous power. Participants were blinded to all other measures.

#### 2.2.5. Statistical analysis

For each trial, mean values for time to completion (hr:min:sec), peak and average power output (W), and VAS responses were computed for the 20 km TT test. Mean values for time to fatigue (hr:min:sec) and VAS responses were computed for the HIEC test. Differences between trials were assessed with a paired samples T-test. Mean values were computed for the OMNI perceived exertion measures in the 20 km TT and analyzed with a two-way repeated measures ANOVA. Pearson correlation coefficients were computed to assess the relationship individually for both supplement and placebo trials with regard to 20 km TT average power, 20 km TT peak power, 20 km TT time to completion, HIEC time to fatigue, and VO2 max. A simple linear regression was performed for significant correlations. A chi-square test of independence was performed to examine the relationship between the order of treatment (supplement first or placebo first) and whether there was improvement between the two trials (yes or no) for the 20 km TT time to completion, 20 km TT average power, 20 km TT peak power, and HIEC time to fatigue. The same statistical approach was used to examine the relationship between the age range (30–39, 40–49, 50–59) and the same test variables. Data were analyzed using SPSS (Version 28.0.1.0) with a significance set at *p* < 0.05.

## 3. Results

### 3.1. 20 km time trial

Peak power (W ± SD) was significantly higher (*p* = .003) during the 20 km TT in the supplement trial compared to the placebo trial (354.27 ± 87.88 and 321.67 ± 63.65, for supplement and placebo trials, respectively), with an average increase in peak power of 11%. There was no significant difference between trials for mean (± SD) 20 km TT time to completion (0:31:50 ± 0:02:16 min and 0:31:46 ± 0:02:04 min for the supplement and placebo trials, respectively; *p* = .316). There was also no significant difference between trials for average power (mean W ± SD) (245.6 ± 48.37 and 246.43 ± 44.29, for supplement and placebo trials, respectively; *p* = .386).

There was no significant difference in average OMNI rating of perceived exertion overall from the beginning until the end of test or at any of the distance points (Table 2). Perceived exertion ratings increased linearly over the execution time of the TT in both the supplement and placebo groups and reached a maximum at the end for both treatments. There was no significant improvement in reported muscular fatigue, overall energy, or ease of pedaling from the supplement trial compared to the placebo trial. There was a significantly lower level of reported muscular soreness (*p*=.049) and greater mental/focus concentration (*p*=.030) from the placebo trial as compared to the supplement trial (Table 3).

**Table 2.** Average OMNI-Cycle scale perceived exertion (mean ± SD).

Treatment	Distance			
	5 km	10 km	15 km	20 km
Supplement	6.39 ± 1.59	7.76 ± 1.07	8.38 ± 1.02	9.47 ± 0.69
Placebo	6.52 ± 1.17	7.55 ± 1.08	8.35 ± 0.69	9.47 ± 0.63

**Table 3.** 20 km time trial test VAS results.

VAS Measurement	Supplement Mean (± SD)	Placebo Mean (± SD)	*p*-value
Muscular fatigue	71.8 (±20.6)	64.0 (±21.0)	0.076
Muscular soreness	50.0 (±32.4)	42.2 (±27.6)	0.049*
Overall energy	60.7 (±23.6)	55.6 (±25.9)	0.447
Ease of pedaling	53.7 (±26.6)	53.2 (±25.2)	0.920
Mental focus/concentration	41.0 (±27.0)	28.0 (±26.0)	0.030*

* Indicates p-value <.05 (two-tailed).

### 3.2. HIEC test

For the HIEC, time to fatigue was significantly greater (*p* = .001) for the supplement trial (0:19:49 ± 0:11:13 min) as compared to the placebo trial (0:14:33 ± 0:09:59 min), with an average increase in time to fatigue of 36.2%. The greatest improvement in time to fatigue was 0:26:28. There was no significant improvement in reported muscular fatigue, overall energy, ease of pedaling, or mental focus/concentration from the supplement trial compared to the placebo trial. There was a significantly lower level of reported muscular soreness with the placebo trial as compared to the supplement trial (*p*=.026) (Table 4).

**Table 4.** High intensity endurance cycling test VAS results.

VAS Measurement	Supplement Mean (± SD)	Placebo Mean (± SD)	*p*-value
Muscular fatigue	79.1 (±25.6)	81.4 (±18.5)	.275
Muscular soreness	65.6 (±29.9)	57.0 (±28.7)	.026*
Overall energy	65.6 (±23.5)	65.8 (±24.7)	.483
Ease of pedaling	73.1 (±24.8)	75.6 (±20.2)	.204
Mental focus/concentration	48.0 (±29.5)	51.0 (±34.0)	.328

* Indicates *p*-value <.05 (two-tailed).

### 3.3. Correlation statistics

**Figure 1.**
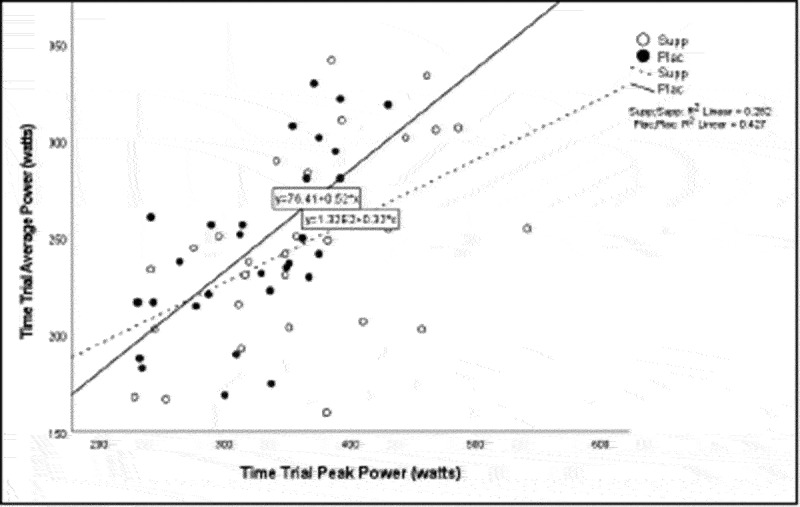
Time trial peak power versus time trial average power.

**Figure 2.**
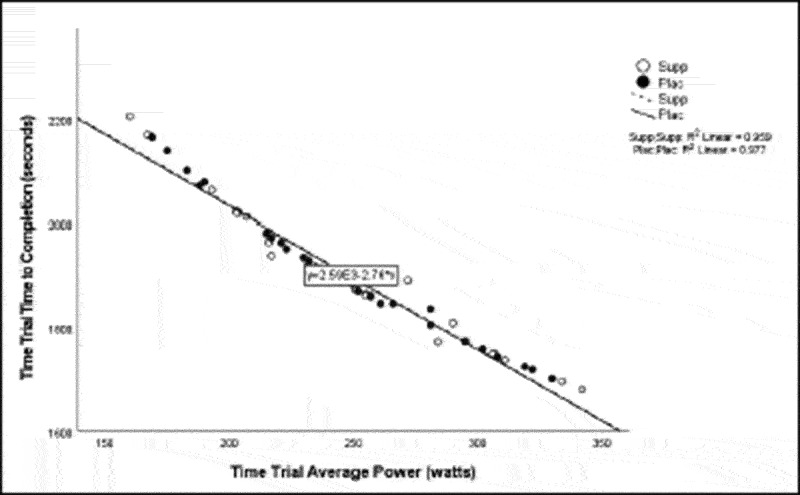
Time trial average power versus time trial time to completion.

**Figure 3.**
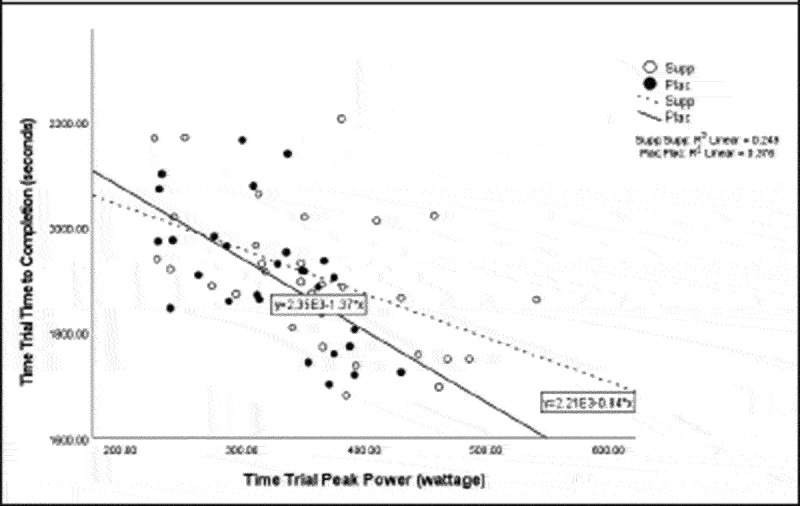
Time trial peak power versus time trial time to completion.

**Figure 4.**
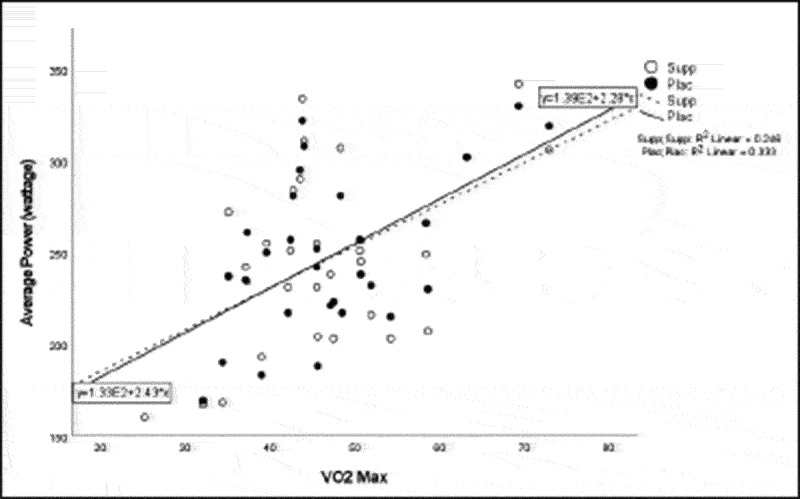
VO_2_ max versus time trial average power.

**Figure 5.**
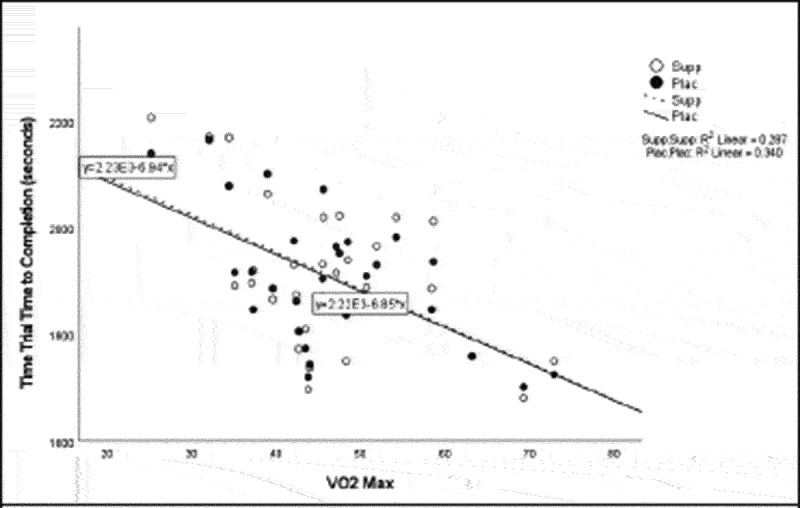
VO_2_ max versus time trial time to completion.

Pearson correlation coefficients were computed to assess the linear relationship between the following variables for all participants for the supplement and placebo trials: 20 km TT average power, 20 km TT peak power, 20 km TT time to completion, HIEC time to fatigue, and VO_2_ max for the supplement trial (Table 5) and placebo trial (Table 6). Simple linear regression was performed for the significant correlations to test if the independent variable (x) significantly predicted the dependent variable (y) (Figures 1–5).

**Table 5.** Correlations between cycling test variables – supplement trial.

	1	2	3	4	5
1. TT Average Power	1.00				
2. TT Peak Power	.531**	1.00			
3. TT Time to Completion	−.979***	−.499**	1.00		
4. HIEC Time to Fatigue	.157	−.066	−.228	1.00	
5. VO_2_ Max	.496**	.299	−.535**	−.210	1.00

** Indicates *p*-value <.005 (two-tailed); *** Indicates *p*-value <.001 (two-tailed).

**Table 6.** Correlations between cycling test variables – placebo trial.

	1	2	3	4	5
1. TT Average Power	1.00				
2. TT Peak Power	.654***	1.00			
3. TT Time to Completion	−.988***	−.613***	1.00		
4. HIEC Time to Fatigue	−.008	−.274	−.054	1.00	
5. VO_2_ Max	.577***	.324	−.583***	−.107	1.00

** Indicates *p*-value <.005 (two-tailed); *** Indicates *p*-value <.001 (two-tailed).

There was no significant relationship between the order of treatment and improvement in variables: TT average power, *X*^*2*^ (1, *N* = 30) = .201, *p* = .654, TT peak power, *X*^*2*^ (1, *N* = 30) = .106, *p* = .745, TT time to completion, *X*^*2*^ (1, *N* = 30) = .089, *p* = .765; *p* = .654; HIEC time to fatigue, *X*^*2*^ (1, *N* = 30) = .106, *p* = .745. There was no significant relationship between age and improvement in variables: TT average power, *X*^*2*^ (2, *N* = 30) = .831, *p* = .660, TT peak power, *X*^*2*^ (2, *N* = 30) = 3.619, *p* = .164, TT time to completion, *X*^*2*^ (2, *N* = 30) = 1.249, *p* = .536; HIEC time to fatigue, *X*^*2*^ (2, *N* = 30) = 2.274, *p* = .321.

## 4. Discussion

It was hypothesized that a supplement containing BCAAs, L-citrulline, and A-GPC would result in improved performance by increasing peak and average power, reducing time to completion, and reducing measures of perceived exertion in a 20 km TT test and increasing time to fatigue and reducing measures of perceived exertion in a follow-on HIEC test. To date, this is the first study examining the potential effects of this specific combination of ingredients. This study also intended to fill gaps in the scientific literature concerning the efficacy of supplementation of amino acids for enhancing performance in trained cyclists by investigating multiple measures of performance with a 20 km cycling TT test and a HIEC, as well as using a chronic loading protocol for administration of the supplement.

### 4.1. 20 km time trial

Participants experienced a significant increase in peak power with the supplement compared to the placebo, with an average increase in peak power of 11%. There was also a moderate positive correlation for VO_2_ max and TT peak power, which supports that VO_2_ max can be accurately predicted from cycling peak power [32]. The observed effects are likely due in part to the BCAAs contained in the supplement. Exercise of strenuous nature increases energy expenditure and subsequent catabolism of muscle proteins and free fatty acids as they help maintain energy levels during exercise after glycogen depletion. Supplementation with BCAAs pre-exercise increases circulating BCAAs and decreases the catabolic effects during exercise [4,24,33,34]. The increase in peak power observed in the present study is in agreement with Kephart et al. [33] who found a 19% increase in peak power in trained cyclists from chronic 10-week supplementation with 12 g of BCAAs (3:1:2; leucine, isoleucine, valine). Manaf et al. [5] also found that acute supplementation with BCAAs (pre-exercise: 0.084 g/kg BW; during exercise: 0.056 g/kg/hr) increased average power output and improved the time to completion by 7% in recreationally active men (untrained cyclists). Crowe et al. [35] found chronic 6-week ingestion of L-leucine (45 mg/kg/d) enhanced short-term power output (10-s upper body power) in trained canoeists. The authors concluded that the increased performance observed was not likely due to reduced central fatigue as they did not observe a significant reduction in the plasma ratio of f-TRP to BCAA. The current data along with the data reported by the aforementioned authors collectively suggest that BCAA supplementation may enhance power output across varying exercise modalities and levels of training.

More research is needed to determine if the benefits from BCAA supplementation are allocated to specific muscles, types of movements, and/or exercise modality. This study did not assess changes in lean tissue mass; therefore, it is unclear if the supplement enhanced muscle functionality in the absence of hypertrophy. Future studies could assess this aspect noninvasively by using ultrasonography to image muscle structure and examine change in the muscle to better understand the effects of supplementation on performance measures [36]. Ultrasonography has been found to be a viable and applicable tool, particularly for measuring changes in the quadriceps muscle [37].

BCAAs are also noted to be of importance for their protein-sparing effect in the period after exercise [34] and for reducing markers of muscle damage and feelings of soreness [38]. Leucine in particular is known for its critical role in muscle protein synthesis as it is needed for maximal activation of mechanistic target of rapamycin complex-1 (mTORC1) signaling, the key regulator of muscle protein synthesis. mTORC1 when active also inhibits autophagy and protein degradation [39]. Thus, in addition to the contribution of energy during the cycling test, it is likely that the observed effect of increased power production is also due in part to the 7-day loading protocol used as daily intake of BCAAs may have contributed to reduced muscle protein breakdown from regular training maintained during the supplementation period [40], which would provide beneficial indirect impact to recovery and contribute to an adaptive response in the individual.

The addition of citrulline is likely to have also played a role in the observed effects in the present study as oral supplementation of citrulline increases arginine, which is the primary substrate for NO biosynthesis, resulting in increased blood flow, oxygen, and nutrient delivery to muscles [11]. Similar to the findings in the present study, Terasawa and Nakada [11] observed an increase in mean power output, pedaling speed per 5 s, and power output per 5 s during a Wingate test after supplementation with 3 g/day of citrulline. The beneficial impact of citrulline supplementation is suggested to be more evident in older athletes due to decreased NO production that occurs with aging [41]. There was no significant correlation between age and improvement in any of the cycling test variables. However, it is possible that any specific improvements based on age were not evident due to the large age range in the study.

As strenuous exercise can create a significant, short-term decrease in free choline in the blood that would inhibit excitation – contraction coupling at the neuromuscular junction [3], it is suggested that A-GPC played a synergistic role with the other ingredients in the supplement in the observed peak power output. The increased peak power finding in the present study is in alignment with findings by Bellar et al. [3] who reported that supplementation with 600 mg of A-GPC for 6 days increased lower body force production, Cruse [18] who found a 12% increase in upper body power output after consuming 6 mg/kg body mass of A-GPC 25-min prior to exercise, and Ziegenfuss et al. [42] who found that supplementation with a single dose of 600 mg of A-GPC 90 min prior to resistance exercise increased peak bench press force by 14%. In addition, A-GPC supplementation has been shown to enhance the release of human growth hormone (HGH) from the pituitary gland. HGH stimulates cell growth and repair, and supplementation has been shown to enhance muscular strength and power in athletes [3,43]. There is the potential that the improved power production was in part related to an increase in HGH over the 7-day supplementation period. Longer duration chronic loading studies are needed to better detect the potential for increases in HGH from A-GPC supplementation to improve performance. Future research should also focus on dosage, timing of consumption, and subject selection to reduce individual variability to more effectively determine beneficial impacts to HGH from A-GPC supplementation.

Previous studies reported increases in average power from BCAA supplementation [33] and L-citrulline supplementation [11]. Despite the lack of significant findings for increase in average power and TT time to completion in the present study, there was a moderately negative correlation for VO_2_ max and TT time to completion suggesting that participants with a higher VO_2_ max had a shorter TT time to completion. The discrepancy in and lack of findings in these variables may have been due to a potential learning effect between the performance testing sessions and the stacked protocol used in the study. These findings are also similar to a study by Kephart et al. [33] who noted an improvement in peak power from a supplement containing 12 g of BCAAs, but no improvement in 4 km TT performance. Greer et al. [4] also found no significant improvement in TT trial time to completion following supplementation with 24.3 g BCAAs. The distinct difference in the cycling distance in these studies, yet similar findings, highlights the opportunity for future research on cycling tests of different durations and intensities to fully understand the benefits to improved power production.

Various studies have found BCAA ingestion before exercise contributes to reduced perceived exertion during exercise [4,5] as has citrulline [11,25,44,45]; however, the present study did not find any significant improvement in OMNI-cycle scale perceived exertion at any of the 20 km distance points or end of test VAS responses. Reduced perceived exertion during exercise has been associated with serum blood Trp:BCAA ratio and central fatigue development [34]. As mechanisms underlying the central fatigue hypothesis are suggested to take an extended duration to manifest (i.e. >1 h) [35], there is the potential that participants had not exercised long enough for the benefits to be notable during the TT test. Similarly, the effectiveness of citrulline on reduced muscular fatigue is likely more evident in longer duration testing protocol as its effects would be less in shorter duration activity where lower levels of ammonia production and lactate would occur. Athletes have a strong pacing strategy and often use perception of fatigue to dictate how much effort to expend during exercise [46]; therefore, it could be expected that participants would have reported similarly regarding perceived exertion upon completion of each testing session.

### 4.2. HIEC test

Participants experienced a significant increase in time to fatigue with the supplement compared to the placebo, with an average increase in time to fatigue of 36.2%. The observed effects are likely attributed to enhanced power production that allowed the participants to cycle at a higher intensity for longer without fatigue. The present study did not assess changes in lean tissue mass; therefore, it is unclear if the supplement enhanced muscle functionality in the absence of hypertrophy. Future studies could assess this aspect noninvasively by using ultrasonography to image muscle structure and examine change in the muscle to better understand the effects of supplementation on performance measures.

The BCAAs contained in the supplement likely improved time to fatigue by serving as an energy substrate contributing to improved peripheral fatigue factors and by delaying central fatigue. Glucose is used as a major energy source during exercise; however, it is suggested that amino acids contribute approximately 5% of energy source during exercise (with ranges estimated up to 20% based on factors such as carbohydrate availability and sex) [47]. Despite this relatively minor role, the additional fuel source during the prolonged exercise in the present study likely contributed to the increased time to fatigue. This observation is in alignment with AbuMoh’d et al. [48] who found acute supplementation of 20 g of BCAAs increased time to exhaustion for 16 male long-distance runners who completed an incremental treadmill test. In the current study, it is more likely that time to fatigue was increased due to delay in central fatigue as shown in previous studies [1,48] with BCAA supplementation improving Trp:BCAA ratio. The present study used VAS responses to assess central fatigue. Central fatigue can display as loss of focus, decreased memory, and slower reaction time, therefore, addition of a reaction time test pre- and post-performance testing in future studies would provide additional data to support effects of BCAA supplementation on central fatigue.

Kephart et al. [33] concluded that BCAA supplementation is more effective in enhancing power-associated variables as compared to endurance performance variables. Therefore, it is likely that citrulline and/or A-GPC contributed to enhanced muscular endurance and delay in peripheral fatigue. This is in alignment with findings in the literature demonstrating improvements to muscular endurance [44,49] and muscular strength [11,50,51] from citrulline supplementation. A-GPC supplementation has also been shown to positively impact muscular strength and power production [3], and the potential for benefits to muscular endurance if choline limitation affects other physiological variables [15]. It is suggested that chronic supplementation of citrulline is likely to have greater performance benefits, specifically in endurance exercise [50]; therefore, future studies should continue to investigate impacts from a chronic loading protocol. To date, studies on performance benefits from A-GPC used markers of performance related to strength training [3] and anaerobic power output [52]. The present study adds valuable information on the role of A-GPC with endurance cycling performance.

There was no improvement in VAS responses with the test supplement. The HIEC test is designed for the participant to go until complete exhaustion; therefore, it was expected that participants would report similarly regarding perceived exertion upon completion of each testing session.

### 4.3. Study limitations and future research

A major strength of this study is that it was a double-blind, placebo-controlled, crossover study. Procedures for dietary intake and exercise patterns were implemented to achieve consistency throughout the study period. Participants were given a diet and activity log to record their food intakes and exercise during the 72 h prior to the first trial and were instructed to repeat the diet and activity pattern for the second trial. Participants were instructed to be as specific as possible, including type and preparation of food and amount of food consumed. Due to variability in detail of the logs, it was not possible to calculate macronutrient intake of the participants. As the diet logs were self-reported, it can only be assumed that the logs were accurate and precise. Future studies should strive to better address this study limitation and consider providing a standard menu to participants with calorie and macronutrient guidance based on factors such as age, height, weight, sex, and activity level to provide greater dietary control rather than allowing participants to record and replicate diet and exercise during the trials

## 5. Conclusions

This study is the first to investigate the effects of supplementation from the combination of BCAAs, L-citrulline, and A-GPC on exercise performance. There was a significant increase in peak power during the 20 km TT test and increased time to fatigue in the HIEC test. These observed improvements would represent a meaningful difference in athletic performance as a slight increase in performance is often what is needed to change the overall outcome in an athletic competition. Since there are multiple ingredients in the supplement, it is not possible to determine which ingredient(s) contributed the most to the ergogenic effect and to what degree. It can be argued that a supplement like this one with ingredients that work both uniquely and synergistically is likely to provide the greatest benefit to exercise performance. Future studies should investigate BCAAs alone versus this combination to determine the amount of synergistic effect from L-citrulline and A-GPC, in addition to chronic versus acute loading protocol and benefits of this supplement on different measures of performance and with various populations (e.g. age ranges, gender, sports, level of training, season of training). Nonetheless, the results from the present study suggest the combination of BCAAs, L-citrulline, and A-GPC may be useful for individuals seeking to improve athletic performance, particularly in cycling and similar disciplines requiring muscular strength and endurance.

## Data Availability

The datasets used and/or analyzed during the present study are available from the corresponding author on reasonable request.
